# Comparison of postprocedural new-onset atrial fibrillation between transcatheter and surgical aortic valve replacement: A systematic review and meta-analysis based on 16 randomized controlled trials

**DOI:** 10.1097/MD.0000000000026613

**Published:** 2021-07-16

**Authors:** Yongmin Ding, Minmin Wan, Hemei Zhang, Chunyu Wang, Zhuoyu Dai

**Affiliations:** a Medical Department; b Outpatient Department, Zhebei Mingzhou hospital, Huzhou, China.

**Keywords:** aortic valve replacement, new-onset atrial fibrillation, transcatheter aortic valve replacement

## Abstract

**Background:**

Presently, transcatheter aortic valve replacement (TAVR) as an effective and convenient intervention has been adopted extensively for patients with severe aortic disease. However, after surgical aortic valve replacement (SAVR) and TAVR, the incidence of new-onset atrial fibrillation (NOAF) is prevalently found. This meta-analysis was designed to comprehensively compare the incidence of NOAF at different times after TAVR and SAVR for patients with severe aortic disease.

**Methods:**

A systematic search of PubMed, Embase, Cochrane Library, and Web of Science up to October 1, 2020 was conducted for relevant studies that comparing TAVR and SAVR in the treatment of severe aortic disease. The primary outcomes were the incidence of NOAF with early, midterm and long term follow-up. The secondary outcomes included permanent pacemaker (PM) implantation, myocardial infarction (MI), cardiogenic shock, as well as mortality and other complications. Two reviewers assessed trial quality and extracted the data independently. All statistical analyses were performed using the standard statistical procedures provided in Review Manager 5.2.

**Results:**

A total of 16 studies including 13,310 patients were identified. The pooled results indicated that, compared with SAVR, TAVR experienced a significantly lower incidence of 30-day/in-hospital, 1-year, 2-year, and 5-year NOAF, with pooled risk ratios (RRs) of 0.31 (95% confidence interval [CI] 0.23–0.41; 5725 pts), 0.30 (95% CI 0.24–0.39; 6321 pts), 0.48 (95% CI 0.38–0.61; 3441 pts), and 0.45 (95% CI 0.37–0.55; 2268 pts) respectively. In addition, TAVR showed lower incidence of MI (RR 0.62; 95% CI 0.40–0.97) and cardiogenic shock (RR 0.34; 95% CI 0.19–0.59), but higher incidence of permanent PM (RR 3.16; 95% CI 1.61–6.21) and major vascular complications (RR 2.22; 95% CI 1.14–4.32) at 30-day/in-hospital. At 1- and 2-year after procedure, compared with SAVR, TAVR experienced a significantly higher incidence of neurological events, transient ischemic attacks (TIA), permanent PM, and major vascular complications, respectively. At 5-year after procedure, compared with SAVR, TAVR experienced a significantly higher incidence of TIA and re-intervention respectively. There was no difference in 30-day, 1-year, 2-year, and 5-year all-cause or cardiovascular mortality as well as stroke between TAVR and SAVR.

**Conclusions:**

Our analysis showed that TAVR was superior to SAVR in decreasing the both short and long term postprocedural NOAF. TAVR was equal to SAVR in early, midterm and long term mortality. In addition, TAVR showed lower incidence of 30-day/in-hospital MI and cardiogenic shock after procedure. However, pooled results showed that TAVR was inferior to SAVR in reducing permanent pacemaker implantation, neurological events, TIA, major vascular complications, and re-intervention.

## 1 Introduction

At present, degenerative aortic valve disease, as one of the most frequent valvular heart disease with a severity ranging from aortic sclerosis slowly progressing to symptomatic severe aortic stenosis (AS), usually requires aortic valve replacement.^[1]^ In patients older than 75 years, AS is present in 12.4% of the population, with severe forms in 3.4% of the elderly.^[2]^ Currently, though surgical aortic valve replacement (SAVR) was a traditional effective method for patients with symptomatic severe AS, transcatheter aortic valve implantation (TAVR) as an effective and convenient intervention has been adopted extensively.

According to the European and American guidelines, symptomatic severe AS requires SAVR or TAVR, with a mean survival of 2 to 3 years in the absence of these procedures.^[3,4]^ TAVR is increasingly used in high and more recently in intermediate-risk population, studies evaluating now the indication even in low-risk population.^[5–8]^ The 2017 American Heart Association Valvular Guidelines^[9,10]^ have given TAVR a Class I recommendation (level of evidence A) for these patients at high or prohibitive surgical risk. For those at intermediate risk, TAVR is considered a reasonable alternative to SAVR,^[7,11]^ with a Class IIA recommendation in the American Heart Association guidelines.^[9,10]^ These decisions should involve a multidisciplinary heart valve team.

However, after SAVR and TAVR, the incidence of new-onset atrial fibrillation (NOAF) is 31% to 64% and 4% to 32%, respectively.^[12,13]^ NOAF is independently associated with adverse events such as stroke, death, and increased length of hospital stay. Increasing the knowledge of predisposing factors, optimal postprocedural monitoring, and prophylactic antiarrhythmic and antithrombotic therapy may reduce the risk of complications secondary to NOAF.^[14]^

However, at present, the incidence of NOAF after SAVR and TAVR has not yet been well studied. Therefore, this meta-analysis was designed to comprehensively compare the incidence of NOAF at different times after TAVR and SAVR for patients with severe aortic disease.

## 2 Methods

### 2.1 Search strategy and study selection

A systematic search of PubMed, Embase, Cochrane Library and Web of Science up to October 1, 2020 was conducted for relevant studies using a search strategy developed by a medical information specialist that involved controlled vocabulary and keywords related to our research question (eg, “aortic stenosis,” “valvular heart disease,” “aortic valve disease”; “transcatheter aortic valve replacement,” “transcatheter aortic valve implantation,” “surgical aortic valve replacement,” “surgical aortic valve implantation,” “TAVR,” “TAVR,” “SAVR,” “SAVI”; “atrial fibrillation,”,“arrhythmia,” and “complication”). The search strategy was limited to English language articles. Two assessors independently screened the titles and abstracts of each study. When a relevant study was identified, its full text was obtained for further evaluation. The full text of related references was also obtained for review.

### 2.2 Criteria for considering studies

We included studies if they met the following criteria: RCTs that compared TAVR with SAVR; studies in which the relevant outcomes of both TAVR and SAVR groups were assessed; and patients who were diagnosed with severe aortic disease.

Studies were excluded if they met the following criteria: experimental trial on animals or a non-human study, non-RCTs, or observational studies; study population included patients with other diseases that would affect outcomes; study reported in the form of an abstract, letter, editorial, expert opinion, review, or case report; or lack of sufficient data or failure to meet the inclusion criteria.

### 2.3 Quality assessment and data extraction

Two reviewers assessed the quality of each RCT using the previously validated 5-point Jadad scale.^[15]^ Studies with scores of 0 to 1 were considered low quality; scores of 2 to 3 were considered moderate quality; scores of ≥4 were considered high quality. In addition, the risk of bias for each studies and the risk of bias across all studies were evaluated and shown with figures generated by RevMan 5.2 software.^[16]^

Baseline characteristics and outcomes from the included studies were extracted using a standardized extraction form. Key study characteristics including study year, sample size, sex, mean age, intervention, follow-up time, and outcomes, were extracted. Data were extracted by one reviewer and then examined for accuracy and completeness by a second reviewer.

### 2.4 Outcome measures

The primary outcomes were the incidence of NOAF with early, midterm and long term follow-up. NOAF was defined as detection of atrial fibrillation (AF) in a patient with no previous known AF.

The secondary outcomes included permanent pacemaker (PM) implantation, myocardial infarction (MI), cardiogenic shock, as well as mortality and other complications.

### 2.5 Data synthesis and statistical methods

The data of comparable outcomes between TAVR and SAVR were combined-analyzed, using the standard statistical procedures provided in RevMan 5.2.^[16]^ Dichotomous data were measured with risk ratio (RR) and continuous variable data were measured with mean difference (MD). The heterogeneity between studies was evaluated by the *χ*^2^-based Q statistical test,^[17]^ with *P*_*h*_ value and *I*^*2*^ statistic, ranging from 0% to 100%, to quantify the effect of heterogeneity. *P*_*h*_ ≤.10 was deemed to represent significant heterogeneity,^[18]^ and pooled estimates were estimated using a random-effect model (the DerSimonian and Laird method ^[19]^). On the contrary, if statistical study heterogeneity was not observed (*P*_*h*_ > .10), a fixed-effects model (the Mantel–Haenszel method ^[20]^) was used. The effects of outcome measures were considered to be statistically significant if pooled RRs with 95% confidence interval (CI) did not overlap with 1 or pooled MDs with 95% CI did not overlap with 0.

This work has been reported in line with Preferred Reporting Items for Systematic Reviews and Meta-Analyses^[21]^ and Assessing the methodological quality of systematic reviews Guidelines.^[22]^ The present study was approved by the Ethics Committee of Lanzhou University First Affiliated Hospital.

## 3 Results

### 3.1 Included studies, study characteristics, and quality assessment

**Figure 1:**
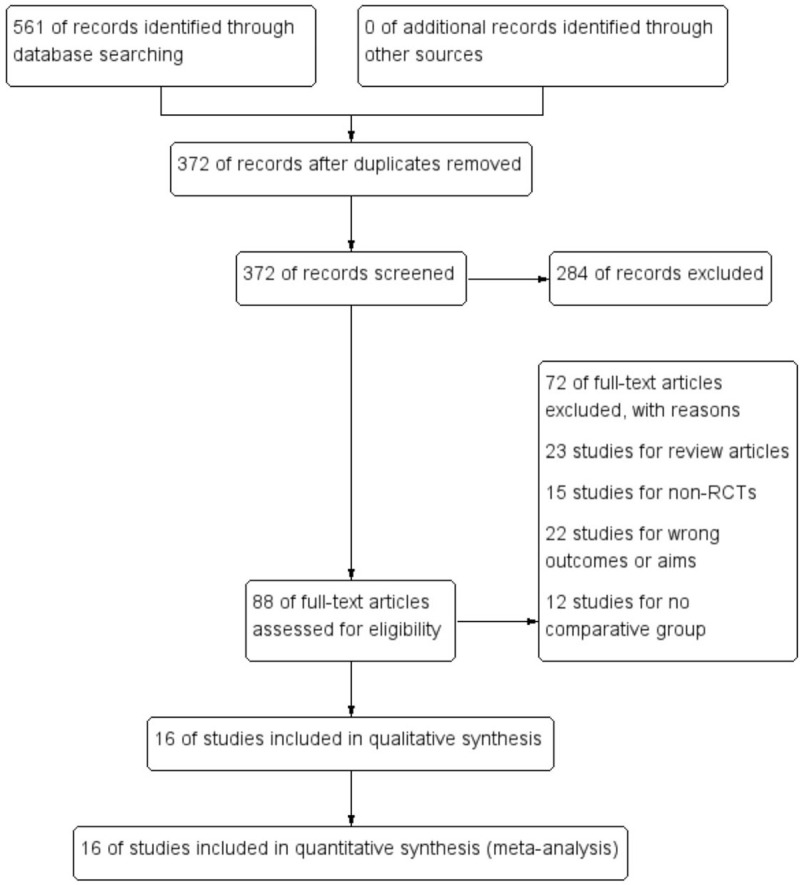
Flow diagram of literature search and selection of included studies for meta-analysis.

At the beginning of the search, a total of 561 records of citations were obtained; 372 of records were reviewed further after duplicates were removed. Via screening the titles and abstracts, 129 studies were excluded preliminarily and then 88 studies were chosen to get full texts for further evaluation. After reading the full texts, 72 studies were excluded further (23 studies for review articles, 15 for non-RCTs, 12 for lack of controls, and 22 for erroneous aims). Eventually, 16 RCTs^[7,8,11,23–35]^ (N = 13,310 participants) were included in this systematic review and meta-analysis. Of these studies, except two studies,^[24,28]^ the others were about multicenter studies. The detailed search process and summary of studies are shown in the study flow diagram (Fig. 1). The other characteristics of each study are shown in Table 1.

**Table 1:** The characteristics of included RCTs for meta-analysis.

		Sample size							
Study	Year	TAVR	SAVR	Age, y (mean ± SD)	STS score (mean ± SD, %)	Location	Follow-up time	Primary outcomes	Jadad score
Jørgensen et al^[24]^	2017	27	25	79 (73–82) 77 (73–79)	2.3 (1.7–2.9) 2.0 (1.8–2.8)	Single-center	12 wk	The incidence and temporal development of NOAF	4
Leon et al^[7]^	2016	1011	1021	81.5 ± 6.7 81.7 ± 6.7	5.8 ± 2.1 5.8 ± 1.9	Multicenter	24 mo	Death from any cause or disabling stroke at 2 y	5
Mack et al^[29]^	2015	348	351	84.1 ± 6.6	11.8 ± 3.3 11.7 ± 3.5	Multicenter	60 mo	All-cause mortality in the ITT population at 1 and 5 y,	4
Mack et al^[8]^	2019	496	454	73.3 ± 5.8 73.6 ± 6.1	1.9 ± 0.7 1.9 ± 0.6	Multicenter	12 mo	Composite of all-cause death, stroke, or rehospitalization at 1 y	4
Makkar et al^[33]^	2020	994	994	81.5 ± 6.7 81.7 ± 6.7	5.8 ± 2.1 5.8 ± 1.9	Multicenter	60 mo	Nonhierarchical composite of death from any cause or disabling stroke at 2 y in the ITT population	4
Miller et al^[27]^	2012	344	313	83.6 ± 6.8 84.4 ± 6.3	11.8 ± 3.3 11.7 ± 3.4	NR	24 mo	All neurologic events and all-cause mortality	4
Motloch et al^[28]^	2012	84	86	81.0 ± 0.7 76.8 ± 0.5	4.43 ± 2.7 3.05 ± 2.4	Single-center	72 h	The incidence of NOAF between TAVR and SAVR	3
Nielsen et al^[26]^	2012	34	36	80 ± 3.6 82 ± 4.4	3.1 ± 1.5 3.4 ± 1.2	Multicenter	3 mo	The composite of all-cause mortality, cerebral stroke and/or RF requiring haemodialysis at 30 days	4
Popma et al^[23]^	2019	725	678	74.1 ± 5.8 73.6 ± 5.9	1.9 ± 0.7 1.9 ± 0.7	Multicenter	12.2 mo	Composite of all-cause death or disabling stroke at 24 mo	4
Reardon et al^[32]^	2015	391	359	83.2 ± 7.1 83.3 ± 6.3	7.3 ± 3.0 7.5 ± 3.3	Multicenter	24.4 mo	The 2-y clinical and echocardiographic outcomes	4
Reardon et al^[34]^	2016	202	181	81.5 ± 7.6 81.2 ± 6.6	5.3 (4.3–6.1) 5.3 (4.1–5.9)	Multicenter	24 mo	All-cause mortality and quality of life through 2 y	4
Reardon et al^[41]^	2017	864	796	79.9 ± 6.2 79.7 ± 6.1	4.4 ± 1.5 4.5 ± 1.6	Multicenter	24 mo	Composite of death from any cause or disabling stroke at 24 mo	5
Serruys et al^[30]^	2018	1660	75.1 ± 6.5 75.4 ± 5.5	2.3 ± 0.5 2.3 ± 0.5	Multicenter	24 mo	Composite of all-cause death or disabling stroke at 24 mo	4	
Søndergaard et al^[31]^	2016	142	134	79.2 ± 4.9 79.0 ± 4.7	2.9 ± 1.6 3.1 ± 1.7	Multicenter	24 mo	The composite rate of death from any cause, stroke, or MI	4
Thyregod et al^[35]^	2015	145	135	79.2 ± 4.9 79.0 ± 4.7	2.9 3.1	Multicenter	12 mo	The composite rate of death from any cause, stroke, or MI at 1 y	4
Thyregod et al^[25]^	2019	280	79.1 ± 4.8	3.0 ± 1.7	Multicenter	60 mo	The rate of all-cause mortality, stroke, or MI	4	

IT = intention-to-treat, MI = myocardial infarction, NOAF = new-onset atrial fibrillation, RF = renal failure, SAVR = surgical aortic valve replacement, SD = standard deviation, STS score = the Society of Thoracic Surgeons score, TAVR = transcatheter aortic valve replacement.

**Figure 2:**
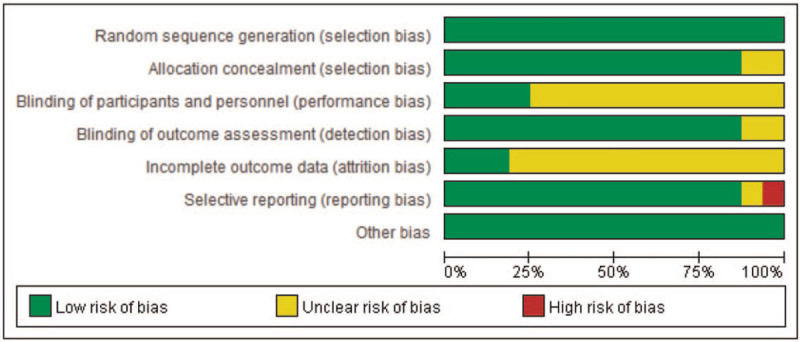
Risk of bias graph: review authors’ judgements about each risk of bias item presented as percentages across all included studies.

**Figure 3:**
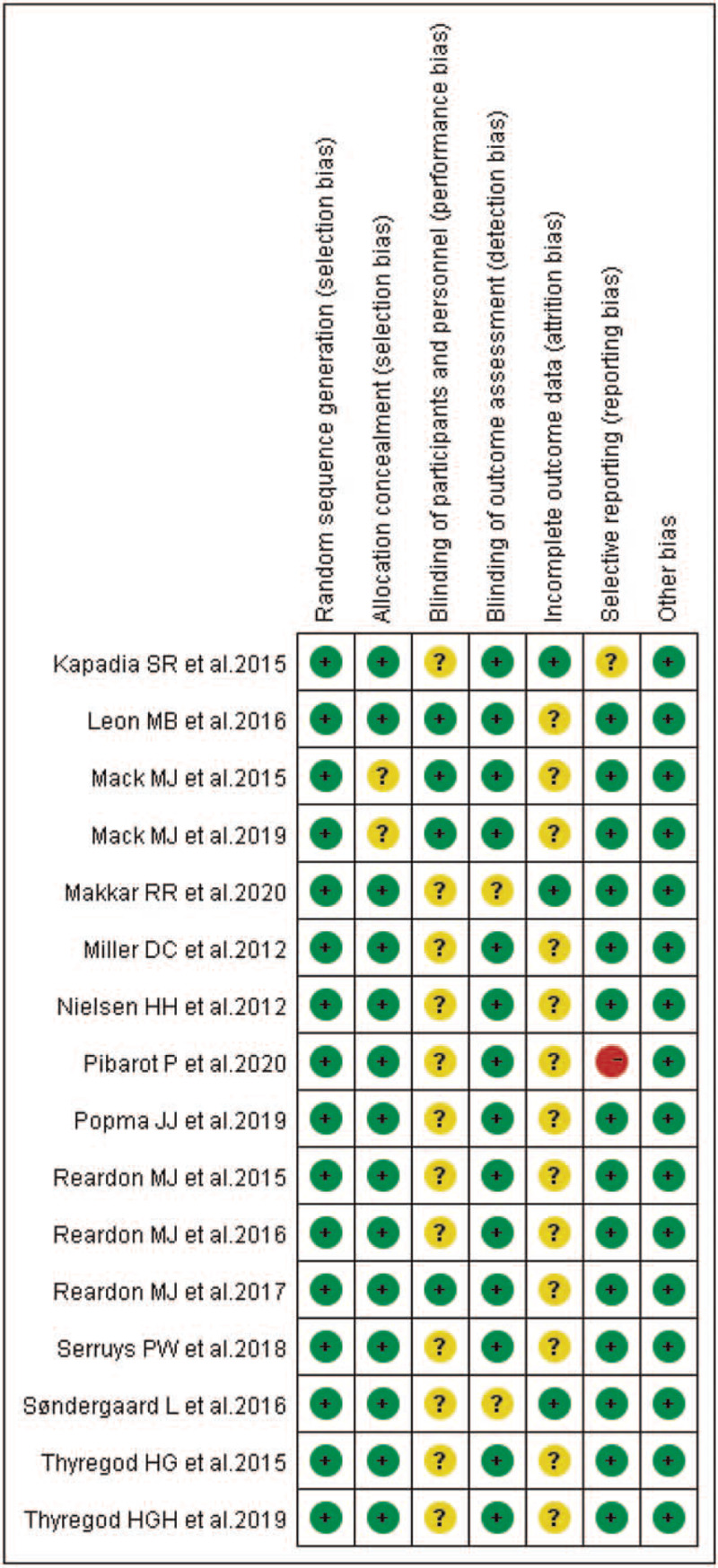
Risk of bias summary: review authors’ judgements about each risk of bias item for each included study.

According to our definitions, there were no low-quality studies included in this analysis. Except Motloch et al (2012)^[28]^ evaluated as moderate quality, the other studies were rated as high quality (93.7%). Additionally, risk-of-bias graphs were generated to further identify the risk of bias of the including studies. The risk of bias for each RCT was presented as percentages across all included studies, and the risk-of-bias item for each included study was displayed (Figs. 2 and 3). The risk-of-bias graphs indicated generally low risk of selection, detection, reporting, and other bias. All studies experienced low risk of bias in “Random sequence generation” item and other bias. A high risk of bias was mainly observed in reporting bias in one study.^[36]^ An unclear risk of bias was mainly observed in performance and attrition bias.

### 3.2 Comparison between TAVR and SAVR regarding to baseline characteristics

We compared the baseline characteristics of both TAVR and SAVR groups with a total of 16 studies (N = 13,310 participants). As Table 2 showing, there was no difference between TAVR and SAVR groups in age (MD −0.06; 95% CI −0.30 to 0.18; 10,423 pts), left ventricular ejection fraction (LVEF) (%) (MD −0.39; 95% CI −0.94 to 0.15; 3986 pts), aortic valve area (cm^2^) (MD 0.02; 95% CI −0.04 to 0.07; 3080 pts), and aortic-valve peak gradient (mmHg) (MD 0.64; 95% CI −1.11 to 2.38; 3080 pts), respectively. In addition, there was also no difference between TAVR and SAVR groups in the proportion of diabetes mellitus, serum creatinine >2 mg/dL, previous stroke, previous transient ischemic attacks (TIA), peripheral vascular disease, previous pacemaker implantation, previous coronary-artery bypass grafting, previous percutaneous coronary intervention, previous myocardial infarction (MI), history of arrhythmia, AF, NYHA Class III/IV, cerebral vascular disease, chronic obstructive pulmonary disease, pulmonary hypertension, and hypertension, respectively. However, significant difference between TAVR and SAVR groups was observed in the proportion of coronary artery disease (CAD) (RR 0.96; 95% CI 0.92–1.0; 5671 pts) and congestive heart failure (MD 0.98; 95% CI 0.97–1.00; 3320 pts).

**Table 2:** The pooled baseline characteristics results of comparison between TAVR and SAVR for severe AS.

		Pooled results			Heterogeneity		
Subgroups	No. of study/pts	RR	95% CI	*P*	*I*^2^	*P*_*h*_	Analytical effect model
DM	7/6772	RR 0.96	0.90–1.03	.25	29%	.21	Fixed-effects model
Serum Cr >2 mg/dL	6/6022	RR 0.88	0.56–1.38	.57	0%	.80	Fixed-effects model
Previous stroke	5/5058	RR 0.88	0.72–1.07	.20	0%	.86	Fixed-effects model
Previous TIA	4/4718	RR 1.09	0.88–1.34	.44	0%	.86	Fixed-effects model
PVD	8/7405	RR 1.0	0.93–1.08	1.00	0%	.97	Fixed-effects model
Previous PM	5/7354	RR 1.0	0.87–1.14	.97	0%	.92	Fixed-effects model
CAD	5/5671	RR 0.96	0.92–1.0	.04	16%	.31	Fixed-effects model
Previous CABG	5/6124	RR 0.94	0.85–1.04	.25	0%	.97	Fixed-effects model
Previous PCI	6/6395	RR 1.0	0.91–1.09	.99	0%	.89	Fixed-effects model
Previous MI	6/6700	RR 1.06	0.93–1.20	.40	0%	.88	Fixed-effects model
CHF	2/3320	RR 0.98	0.97–1.00	.02	0%	.64	Fixed-effects model
History of arrhythmia	2/3320	RR 1.01	0.92–1.12	.79	0%	1.0	Fixed-effects model
AF	7/7271	RR 0.96	0.89–1.04	.32	2%	.41	Fixed-effects model
NYHA Class III/IV	7/7358	RR 1.01	0.96–1.06	.66	50%	.06	Random-effect model
CVD	4/2358	RR 0.97	0.81–1.17	.78	0%	.76	Fixed-effects model
COPD	5/3092	RR 0.91	0.80–1.03	.14	0%	.74	Fixed-effects model
LVEF (%)	5/3986	MD–0.39	−0.94–0.15	.16	0%	.95	Fixed-effects model
Aortic valve area, cm^2^	4/3080	MD 0.02	−0.04–0.07	.51	91%	<.0001	Random-effect model
Aortic-valve peak gradient, mmHg	4/3080	MD 0.64	−1.11–2.38	.48	63%	.05	Random-effect model
PH	2/1563	RR 1.02	0.88–1.19	.76	0%	.54	Fixed-effects model
Hypertension	4/4091	RR 1.01	0.99–1.04	.23	20%	.36	Fixed-effects model

AF = atrial fibrillation, AS = aortic stenosis, CABG = coronary-artery bypass grafting, CAD = coronary artery disease, CHF = congestive heart failure, CI = confidence interval, COPD = chronic obstructive pulmonary disease, Cr = creatinine, CVD = cerebral vascular disease, DM = diabetes mellitus, LVEF = left ventricular ejection fraction, MI = myocardial infarction, PCI = percutaneous coronary intervention, PH = pulmonary hypertension, PM = pacemaker, PVD = peripheral vascular disease, RR = risk ratio, SAVR = surgical aortic valve replacement, TAVR = transcatheter aortic valve replacement, TIA = transient ischemic attacks.

### 3.3 NOAF between TAVR and SAVR

**Figure 4:**
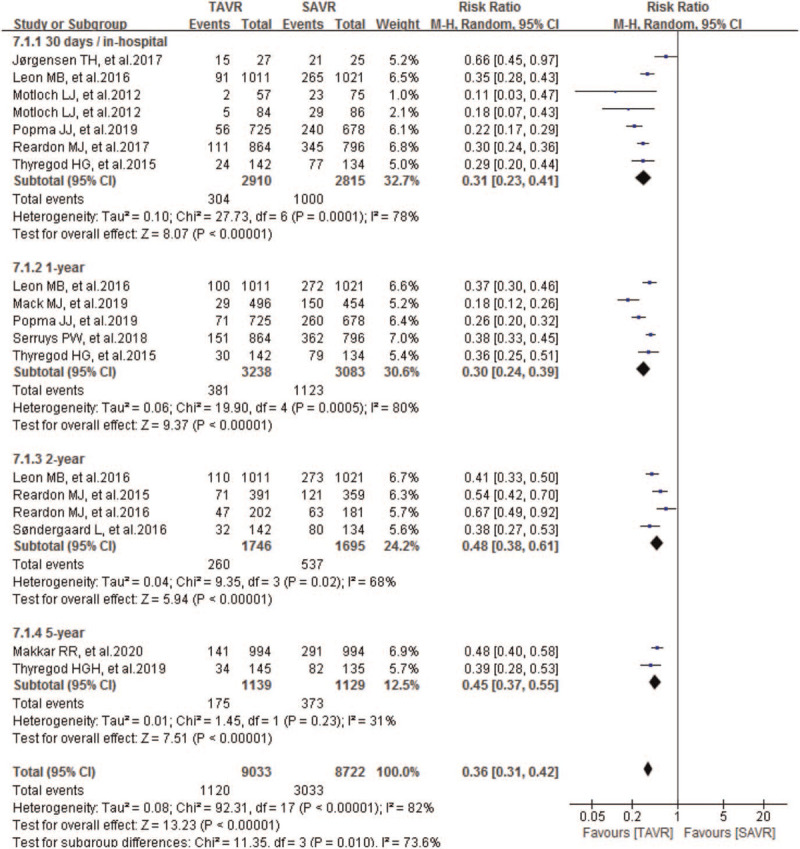
Forest plot of comparison between TAVR and SAVR for severe AS regarding to 30-day/in-hospital, 1-year, 2-year, and 5-year NOAF. AS = aortic stenosis, NOAF = new-onset atrial fibrillation, TAVR = transcatheter aortic valve replacement.

Seven studies compared 30-day/in-hospital NOAF between TAVR and SAVR groups. The incidence of 30-day**/**in-hospital NOAF was 10.4% (304/2910 patients) in TAVR group and 35.5% (1000/2815 patients) in SAVR group. As shown in Figure 4, pooled results showed significant reduction of 30-day/in-hospital NOAF in TAVR than SAVR groups, and the incidence of 30-day/in-hospital NOAF in TAVR was only one-third of SAVR, with a pooled RR of 0.31 (95% CI 0.23–0.41; *P* < .00001; 5725 pts).

The incidence of 1-year NOAF was 11.8% (381/3238 patients) in TAVR group and 36.4% (1123/3083 patients) in SAVR group. The pooled results also showed significant reduction of 1-year NOAF in TAVR than SAVR groups, and the incidence of 1-year NOAF in TAVR was only one-third of SAVR, with a pooled RR of 0.30 (95% CI 0.24–0.39; *P* < .00001; 6321 pts) (Fig. 4). For the incidence of 2-year NOAF, we found similar significance between TAVR and SAVR. The incidence of 2-year NOAF was 14.9% (260/1746 patients) in TAVR group and 31.7% (537/1695 patients) in SAVR group. The pooled results showed a significant reduction of 2-year NOAF in TAVR than SAVR groups, and the incidence of 2-year NOAF in TAVR was only half of SAVR, with a pooled RR of 0.48 (95% CI 0.38–0.61; *P* < .00001; 3441 pts). Similarly, compared with SAVR, TAVR also showed priority in decreasing 5-year NOAF, with a pooled RR of 0.45 (95% CI 0.37–0.55; *P* < .00001; 2268 pts) (Fig. 4).

We displayed the incidence of NOAF between TAVR and SAVR over the following time (Table S1). We could observe that the incidence of NOAF in TAVR showed a slight increasing tendency from 30-day/in-hospital to 5-year follow up time. However, SAVR showed a stable incidence of NOAF over the following time.

### 3.4 The 30-day outcomes between TAVR and SAVR

Six studies compared 30-day mortality of patients with severe AS between TAVR and SAVR groups. Pooled results showed no significant difference in the incidence of 30-day all-cause and CV mortality between TAVR and SAVR groups, with pooled RRs of 0.87 (95% CI 0.65–1.16; *P* = .34; 6098 pts) and 1.04 (95% CI 0.71–1.51; *P* = .85; 4038 pts), respectively. Similarly, compared with SAVR, TAVR showed noninferiority in the following 30-day outcomes: stroke, TIA, life-threatening bleeding, neurological events, endocarditis, CAD, re-intervention, and re-hospitalization (Table 3). In addition, one study also showed noninferiority between TAVR and SAVR in 30-day leakage, cardiac perforation, and LVEF. However, compared with SAVR, TAVR experienced a significantly lower incidence of myocardial infarction (MI) (RR 0.62; 95% CI 0.40–0.97; 5441 pts), cardiogenic shock (RR 0.34; 95% CI 0.19–0.59; 1936 pts), acute kidney injury (AKI) > stage 2 (RR 0.37; 95% CI 0.25–0.54; 5371 pts), but higher incidence of permanent pacemaker implantation (RR 3.16; 95% CI 1.61–6.21; 5441 pts) and major vascular complications (RR 2.22; 95% CI 1.14–4.32; 5371 pts), respectively (Table 3).

**Table 3:** The pooled results of comparison between TAVR and SAVR for severe AS regarding to the 30-day outcomes.

		Pooled results			Heterogeneity		
Subgroups	No. of study/pts	RR	95% CI	*P*	*I*^2^	*P*h	Analytical effect model
Myocardial infarction	5/5441	0.62	0.40–0.97	.04	0%	.79	Fixed-effects model
Cardiogenic shock	2/1936	0.34	0.19–0.59	.0002	0%	.64	Fixed-effects model
AKI >2	4/5371	0.37	0.25–0.54	<.0001	0%	.64	Fixed-effects model
Permanent PM	5/5441	3.16	1.61–6.21	.0008	90%	<.0001	Random-effect model
Major vascular complications	4/5371	2.22	1.14–4.32	.02	77%	.004	Random-effect model
All-cause mortality	6/6098	0.87	0.65–1.16	.34	8%	.36	Fixed-effects model
CV mortality	4/4038	1.04	0.71–1.51	.85	0%	.75	Fixed-effects model
Stroke	5/5441	0.82	0.64–1.04	.10	0%	.42	Fixed-effects model
TIA	5/5441	1.50	0.85–2.66	.16	0%	.66	Fixed-effects model
Bleeding	5/5441	0.51	0.20–1.28	.15	96%	<.0001	Random-effect model
Neurological events	2/2308	0.99	0.72–1.37	.96	0%	.94	Fixed-effects model
Endocarditis	3/3711	1.57	0.21–11.80	.66	0%	.61	Fixed-effects model
CAD	3/5095	1.37	0.60–3.16	.45	13%	.32	Fixed-effects model
Reintervention	3/5095	2.66	1.01–7.00	.05	20%	.29	Fixed-effects model
Rehospitalization	3/5095	0.85	0.66–1.11	.24	46%	.16	Fixed-effects model

AKI = acute kidney injury, AS = aortic stenosis, CAD = coronary artery disease, CI = confidence interval, CV = cardiovascular, LVEF = left ventricular ejection fraction, NOAF = new-onset atrial fibrillation, PM = pacemaker, RF = renal failure, RR = risk ratio, SAVR = surgical aortic valve replacement, TAVR = transcatheter aortic valve replacement, TIA = transient ischemic attacks.

### 3.5 The 1-year outcomes between TAVR and SAVR

Ten studies compared the 1-year mortality between TAVR and SAVR groups. Our pooled results also showed non-inferiority in the incidence of 1-year all-cause and CV mortality of TAVR when compared to SAVR, with pooled RRs of 0.94 (95% CI 0.84–1.06; *P* = .33; 9790 pts) and 0.91 (95% CI 0.76–1.09; *P* = .30; 7277 pts), respectively. Similarly, compared with SAVR, TAVR showed noninferiority in the following 1-year outcomes: stroke, reintervention, MI, endocarditis, re-hospitalization, aortic regurgitation, and CAD (Table 4). In addition, one study also showed noninferiority between TAVR and SAVR in 1-year cardiac perforation, renal failure and LVEF. However, compared with SAVR, TAVR experienced a significantly lower incidence of life-threatening bleeding (RR 0.41; 95% CI 0.24–0.68; 6744 pts), all stage AKI (RR 0.44; 95% CI 0.25–0.77; 4642 pts), AKI > stage 2 (RR 0.56; 95% CI 0.40–0.77; 6045 pts), but higher incidence of neurological events (RR 3.01; 95% CI 1.72–5.27; 6755 pts), TIA (RR 1.44; 95% CI 1.07–1.95; 8680 pts), major vascular complications (RR 2.23; 95% CI 1.19–4.18; 5794 pts), and permanent pacemaker implantation (RR 2.32; 95% CI 1.36–3.95; 7020 pts), respectively (Table 4).

**Table 4:** The pooled results of comparison between TAVR and SAVR for severe AS regarding to the 1-year outcomes.

		Pooled results			Heterogeneity		
Subgroups	No. of study/pts	RR	95% CI	*P*	*I*^2^	*P*_*h*_	Analytical effect model
Bleeding	5/6744	0.41	0.24–0.68	.0007	93%	<.0001	Random-effect model
All AKI	3/4642	0.44	0.25–0.77	.004	68%	.05	Random-effect model
AKI >stage 2	4/6045	0.56	0.40–0.77	.0004	49%	.12	Fixedeffects model
Cardiogenic shock	1/1660	0.32	0.16–0.65	.002			
Neurological events	4/6755	3.01	1.72–5.27	.0001	0%	.46	Fixed-effects model
TIA	7/8680	1.44	1.07–1.95	.02	0%	.88	Fixed-effects model
Major vascular complications	4/5794	2.23	1.19–4.18	.01	83%	.0006	Random-effect model
Permanent PM	6/7020	2.32	1.36–3.95	.002	91%	<.0001	Random-effect model
All-cause mortality	10/9790	0.94	0.84–1.06	.33	0%	.65	Fixed-effects model
CV mortality	6/7277	0.91	0.76–1.09	.30	0%	.52	Fixed-effects model
Stroke	7/8680	0.89	0.75–1.06	.18	38%	.14	Fixed-effects model
Reintervention	3/3968	0.96	0.78–1.18	.67	0%	.42	Fixed-effects model
MI	7/8680	0.91	0.67–1.23	.53	0%	.64	Fixed-effects model
Endocarditis	5/6070	0.82	0.42–1.58	.55	0%	.55	Fixed-effects model
Rehospitalization	6/8404	0.94	0.75–1.18	.60	64%	.02	Random-effect model
Aortic regurgitation	2/1852	1.72	0.88–3.34	.11	0%	.65	Fixed-effects model
CAD	2/3435	1.19	0.49–2.88	.70	36%	.21	Fixed-effects model

AKI = acute kidney injury, AS = aortic stenosis, CAD = coronary artery disease, CI = confidence interval, CV = cardiovascular, LVEF = left ventricular ejection fraction, MI = myocardial infarction, NOAF = new-onset atrial fibrillation, PM = pacemaker, RF = renal failure, RR = risk ratio, SAVR = surgical aortic valve replacement, TAVR = transcatheter aortic valve replacement, TIA = transient ischemic attacks.

### 3.6 The 2-year outcomes between TAVR and SAVR

Six studies compared the 2-year mortality between TAVR and SAVR groups. Our pooled results also showed non-inferiority in the incidence of 2-year all-cause and CV mortality of TAVR when compared to SAVR, with pooled RRs of 0.92 (95% CI 0.83–1.03; *P* = .16; 5758 pts) and 0.87 (95% CI 0.74–1.02; *P* = .09; 5101 pts), respectively. Similarly, compared with SAVR, TAVR showed noninferiority in the following 2-year outcomes: stroke, MI, life-threatening bleeding, and all stage AKI (Table 5). In addition, one study also showed noninferiority between TAVR and SAVR in 2-year endocarditis and CAD. However, compared with SAVR, TAVR experienced a significantly higher incidence of neurological events (RR 1.26; 95% CI 1.02–1.57; 2965 pts), TIA (RR 1.58; 95% CI 1.14–2.17; 5375 pts), permanent pacemaker implantation (RR 2.61; 95% CI 1.36–5.00; 3441 pts), rehospitalization (RR 1.25; 95% CI 1.06–1.46; 3692 pts), major vascular complications (RR 2.38; 95% CI 1.26–4.49; 3165 pts) and reintervention (RR 3.22; 95% CI 1.64–6.29; 3692 pts), respectively (Table 5).

**Table 5:** The pooled results of comparison between TAVR and SAVR for severe AS regarding to the 2-year outcomes.

		Pooled results			Heterogeneity		
Subgroups	No. of study/pts	RR	95% CI	*P*	*I*^2^	*P*_*h*_	Analytical effect model
Permanent PM	4/3441	2.61	1.36–5.00	.004	90%	<.00001	Random-effect model
Rehospitalization	2/3692	1.25	1.06–1.46	.007	0%	.41	Fixed effects model
Major vascular complications	3/3165	2.38	1.26–4.49	.007	58%	.09	Random-effect model
Neurological events	3/2965	1.26	1.02–1.57	.04	0%	.47	Fixed effects model
TIA	5/5375	1.58	1.14–2.17	.006	0%	.97	Fixed effects model
Reintervention	2/3692	3.22	1.64–6.29	.0006	0%	.62	Fixed effects model
All-cause mortality	6/5758	0.92	0.83–1.03	.16	34%	.18	Fixed effects model
CV mortality	5/5101	0.87	0.74–1.02	.09	0%	.55	Fixed effects model
Stroke	5/5101	0.85	0.71–1.02	.09	14%	.33	Fixed effects model
MI	4/4718	0.98	0.71–1.36	.90	0%	.85	Fixed effects model
Bleeding	3/3165	0.56	0.31–1.00	.05	96%	<.00001	Random-effect model
All AKI	3/3165	0.63	0.31–1.30	.21	70%	.04	Random-effect model

AKI = acute kidney injury, AS = aortic stenosis, CAD = coronary artery disease, CI = confidence intervals, CV = cardiovascular, NOAF = new-onset atrial fibrillation, RR = risk ratio, SAVR = surgical aortic valve replacement, TAVR = transcatheter aortic valve replacement, TIA = transient ischemic attacks.

### 3.7 The 5-year outcomes between TAVR and SAVR

Five studies compared the 5-year mortality between TAVR and SAVR groups. Our pooled results indicated non-inferiority in the 5-year all-cause and CV mortality of TAVR when compared to SAVR, with pooled RRs of 1.01 (95% CI 0.78–1.31; *P* = .95; 3325 pts) and 0.95 (95% CI 0.67–1.33; *P* = .75; 3325 pts), respectively. Similarly, when compared with SAVR, TAVR showed noninferiority in the following 5-year outcomes: stroke, rehospitalization, MI, endocarditis and permanent pacemaker implantation (Table 6). In addition, one study also showed noninferiority between TAVR and SAVR in 5-year neurological events and renal failure. However, compared with SAVR, TAVR experienced a significantly higher incidence of TIA (RR 1.50; 95% CI 1.04–2.17; 2967 pts) and re-intervention (RR 3.40; 95% CI 1.47–7.85; 2268 pts), respectively (Table 6).

**Table 6:** The pooled results of comparison between TAVR and SAVR for severe AS regarding to the 5-year outcomes.

		Pooled results			Heterogeneity		
Subgroups	No. of study/pts	RR	95% CI	*P*	*I*^2^	*P*_*h*_	Analytical effect model
TIA	3/2967	1.50	1.04–2.17	.03	0%	.88	Fixed effects model
Major vascular complications	1/699	2.95	1.64–5.32	.0003			
Reintervention	2/2268	3.40	1.47–7.85	.004	0%	.86	Fixed effects model
All-cause mortality	4/3325	1.01	0.78–1.31	.95	93%	<.00001	Random-effect model
CV mortality	4/3325	0.95	0.67–1.33	.75	92%	<.00001	Random-effect model
Stroke	4/3325	1.13	0.93–1.36	.22	0%	.70	Fixed effects model
Rehospitalization	3/3045	0.99	0.52–1.91	.98	97%	<.00001	Random-effect model
MI	3/2967	1.20	0.90–1.58	.21	49%	.14	Fixed effects model
Endocarditis	3/2967	1.40	0.89–2.20	.14	0%	.64	Fixed effects model
Permanent PM	3/2967	1.94	0.85–4.40	.11	90%	<.0001	Random-effect model
Neurological events	1/1988	1.24	1.00–1.53	.05			

AS = aortic stenosis, CI = confidence intervals, CV = cardiovascular, MI = myocardial infarction, RF = renal failure, RR = risk ratio, SAVR = surgical aortic valve replacement, TAVR = transcatheter aortic valve replacement, TIA = transient ischemic attacks.

## 4 Discussion and conclusions

Aortic stenosis is one of the most common valvular problems associated with significant morbidity and mortality in the United States.^[37,38]^ Before TAVR therapy, SAVR was considered the gold standard to improve the prognosis.^[39]^ At present, TAVR has become a valuable therapeutic standard for patients with symptomatic severe aortic stenosis,^[40]^ that was traditionally envisioned to be a treatment option in high-risk surgical candidates.^[41]^ In addition, the encouraging results derived from numerous randomized trials and observational registries corroborate TAVR as a reliable alternative to conventional SAVR in high-risk and intermediate-risk patients and demonstrates a future potential even to moderate to mild risk patients.

At present, several meta-analyses explored the efficacy of TAVR for patients with symptomatic severe aortic stenosis^[6,42–49]^ and found no difference in all-cause mortality or stroke between TAVR and SAVR. However, SAVR and TAVI are associated with a number of different complications including bleeding, vascular injury, and thromboembolism—particularly stroke and arrhythmia. Arrhythmias associated with these interventions are primarily NOAF and conduction disturbances, which may require antiarrhythmic medication, anticoagulant therapy, and/or a need for permanent pacemaker, as well as increasing the length of hospital stay. Thus, the present meta-analysis was designed to comprehensively compare the incidence of NOAF at different times after TAVR and SAVR for patients with severe aortic disease.

Our pooled analysis of 13,310 patients showed that, compared with SAVR, TAVR experienced a significantly lower incidence of 30-day/in-hospital, 1-year, 2-year, and 5-year NOAF, respectively. In addition, TAVR showed lower incidence of MI and cardiogenic shock, but higher incidence of permanent PM and major vascular complications at 30-day/in-hospital. At 1- and 2-year after procedure, compared with SAVR, TAVR experienced a significantly higher incidence of neurological events, TIA, permanent PM, and major vascular complications, respectively. At 5 years after procedure, compared with SAVR, TAVR experienced a significantly higher incidence of TIA and re-intervention respectively. There was no difference in 30-day, 1-year, 2-year, and 5-year all-cause or cardiovascular mortality as well as stroke between TAVR and SAVR. In addition, we could observe that the incidence of NOAF in TAVR showed a slight increasing tendency from 30-day/in-hospital to 5-year follow up time. However, SAVR showed a stable incidence of NOAF over the following time. Conversely, the incidence of permanent PM in SAVR showed an increasing tendency from 30-day/in-hospital to 5-year follow-up time. However, TAVR showed a stable incidence of permanent PM over the following time (see Table S1, supplemental digital content, which illustrates the outcomes of TAVR and SAVR over time).

In the PARTNER trial by Smith et al,^[50]^ patients were randomized to either TAVR with the ESV or SAVR. Not excluding patients with a baseline history of AF, they found a significant difference in the development of NOAF after TAVR and SAVR (9% vs 16% of patients, respectively). Adams et al^[51]^ reported that NOAF or worsening preprocedural AF were significantly more common after SAVR when compared with MCV-TAVI (31% vs 12% of randomized patients, respectively). Unfortunately, there are currently no randomized studies comparing the MCV with the ESV that report the incidence of NOAF.

The incidence of NOAF after SAVR is generally found to be higher than that after TAVR. Many possible factors may result in this discrepancy in the incidence of NOAF between TAVR and SAVR. More serious inflammatory response after SAVR may be one main factor. Inflammation has previously been reported to increase the AF burden and predispose to NOAF after coronary bypass surgery.^[52]^ A similar inflammatory response after the surgical trauma of SAVR might temporarily induce NOAF. Furthermore, diuretics have been associated with an increased risk of NOAF in patients with hypertension potentially because of hypokalemia^[53]^; perhaps, the high doses of diuretics used during the immediate postoperative days after extracorporeal circulation could play a role in the initial high rate of NOAF after SAVR.

There existed several limitations in our work. First, the NOAF detection may exist inconsistency in each included studies which may impact the incidence of NOAF. NOAF detection is often done by continuous monitoring with varying duration ranging between the first 3 to 7 days after the procedure or limited to the length of hospital stay, with NOAF defined as a recorded AF episode lasting >30 seconds or 10 minutes. Furthermore, there is the risk of overestimating the incidence of NOAF. The exclusion of patients with preprocedural AF is often based on a history of previous known AF or short preprocedural screening. As the prevalence of preprocedural AF is high in patients undergoing SAVR and TAVI and AF can be asymptomatic, there is a risk that detected NOAF in some patients is actually the unmasking of preprocedurally unknown AF. Third, the appearance of AF always changes over time. Amat-Santos et al reported that 41% of NOAF occurred within 24 hours, 22% between 24 and 48 hours, 18% between 48 and 72 hours, and 18% occurred >72 hours after TAVI with the ESV. NOAF was reported from the first postprocedural day after SAVR and with the highest incidence after 3 days; however, the study was limited by a postprocedural monitoring period of only 3 days.^[52]^ Finally, the sensitivity of AF detection significantly influenced the incidence of NOAF in each study which failed to unify this and may lead to any bias. Charitos et al reported that the sensitivity of AF detection with intermittent rhythm monitoring was lower when compared to continuous monitoring.^[53]^ Continuous long-term monitoring with implantable loop recorders could be a new helpful clinical tool in detecting and describing NOAF and assessing therapeutic response to NOAF treatment.^[54,55]^

TAVR and SAVR are the only definitive treatments for severe AS; both interventions improve prognosis and symptoms.^[56]^ TAVR, and to a greater degree SAVR, carries a risk of developing NOAF.^[57,58]^ This arrhythmia has significant health, economic, and clinical implications, because the length of hospital stay and the risk of stroke and mortality are increased.^[59]^ Future studies identifying predictive factors for postprocedural NOAF will help in selecting high-risk patients who might benefit from prophylactic antiarrhythmic therapy or surgery.

In conclusion, our analysis showed that TAVR was superior to SAVR in decreasing the both short and long term postprocedural NOAF. TAVR was equal to SAVR in early, midterm and long term mortality. In addition, TAVR showed lower incidence of 30-day/in-hospital MI and cardiogenic shock after procedure. However, pooled results showed that TAVR was inferior to SAVR in reducing permanent pacemaker implantation, neurological events, TIA, major vascular complications, and re-intervention.

## Author contributions

The authors on this paper all participated in study design. All authors read, critiqued and approved the manuscript revisions as well as the final version of the manuscript. Also, all authors participated in a session to discuss the results and consider strategies for analysis and interpretation of the data before the final data analysis was performed and the manuscript written. All authors have the appropriate permissions and rights to the reported data.

**Conceptualization:** Yongmin Ding, Hemei Zhang.

**Formal analysis:** Zhuoyu Dai, Hemei Zhang.

**Methodology:** Minmin Wan, Hemei Zhang.

**Software:** Minmin Wan, Chunyu Wang, Hemei Zhang.

**Writing – original draft:** Minmin Wan, Chunyu Wang, Zhuoyu Dai, Hemei Zhang.

**Writing – review & editing:** Yongmin Ding, Chunyu Wang, Zhuoyu Dai.

## Supplementary Material

Supplemental Digital Content
