# Supplementary material for: Comparison of postprocedural new-onset atrial fibrillation between transcatheter and surgical aortic valve replacement: A systematic review and meta-analysis based on 16 randomized controlled trials
Source: Medicine (Baltimore). 2021 Jul 16;100(28):e26613. doi: 10.1097/MD.0000000000026613 (PMC8284731; doi:10.1097/MD.0000000000026613)
Supplement: Supplemental Digital Content [file medi-100-e26613-s001.docx]

**Table S1**. The outcomes of TAVR and SAVR over time.

| Outcomes | 30-day/in-hospital | | 1-year | | 2-year | | 5-year | |
| --- | --- | --- | --- | --- | --- | --- | --- | --- |
| NOAF | Incidence (%) | RR (95% CI) | Incidence (%) | RR (95% CI) | Incidence (%) | RR (95% CI) | Incidence (%) | RR (95% CI) |
| TAVR | 10.4% | 0.31 (0.23 ‒ 0.41) | 11.8% | 0.30 (0.24 ‒ 0.39) | 14.9% | 0.48 (0.38 ‒ 0.61) | 15.4% | 0.45 (0.37 ‒ 0.55) |
| SAVR | 35.5% |  | 36.4% |  | 31.7% |  | 33.0% |  |
| Permanent PM |  |  |  |  |  |  |  |  |
| TAVR | 16.9% | 2.96 (1.57 ‒ 5.59) | 16.4% | 2.32 (1.36 ‒ 3.95) | 18.4% | 2.61 (1.36 ‒ 5.00) | 15.1% | 1.94 (0.85 ‒ 4.40) |
| SAVR | 6.1% |  | 7.0% |  | 9.5% |  | 9.9% |  |
| MI |  |  |  |  |  |  |  |  |
| TAVR | 1.1% | 0.62 (0.40 ‒ 0.97) | 1.8% | 0.91 (0.67 ‒ 1.23) | 2.9% | 0.98 (0.71 ‒ 1.36) | 6.7% | 1.20 (0.90 ‒ 1.58) |
| SAVR | 1.8% |  | 2.0% |  | 3.0% |  | 5.6% |  |
| Cardiogenic shock |  |  |  |  |  |  |  |  |
| TAVR | 1.6% | 0.34 (0.19 ‒ 0.59) | 1.2% | 0.32 (0.16 ‒ 0.65) |  |  |  |  |
| SAVR | 4.7% |  | 3.6% |  |  |  |  |  |
| Major vascular complications |  |  |  |  |  |  |  |  |
| TAVR | 6.2% | 2.22 (1.14 ‒ 4.32) | 7.0% | 2.32 (1.19 ‒ 4.18) | 8.0% | 2.38 (1.26 ‒ 4.49) | 11.8% | 2.95 (1.64 ‒ 5.32) |
| SAVR | 3.1% |  | 3.5% |  | 4.2% |  | 4.0% |  |
| AKI > 2 |  |  |  |  |  |  |  |  |
| TAVR | 1.3% | 0.37 (0.25 ‒ 0.54) | 1.8% | 0.56 (0.40 ‒ 0.77) | 3.4% | 0.63 (0.31 ‒ 1.30) |  |  |
| SAVR | 3.6% |  | 3.3% |  | 5.8% |  |  |  |
| TIA |  |  |  |  |  |  |  |  |
| TAVR | 1.0% | 1.50 (0.85 ‒ 2.66) | 2.4% | 1.44 (1.07 ‒ 1.95) | 3.4% | 1.58 (1.14 ‒ 2.17) | 4.6% | 1.50 (1.04 ‒ 2.17) |
| SAVR | 0.6% |  | 1.6% |  | 2.2% |  | 3.0% |  |
| Reintervention |  |  |  |  |  |  |  |  |
| TAVR | 0.6% | 2.66 (1.01 ‒ 7.00) | 8.0% | 0.96 (0.78 ‒ 1.18) | 2.0% | 3.22 (1.64 ‒ 6.29) | 2.1% | 3.40 (1.47 ‒ 7.85) |
| SAVR | 0.2% |  | 8.4% |  | 0.6% |  | 0.6% |  |
| Neurological events |  |  |  |  |  |  |  |  |
| TAVR | 5.9% | 0.99 (0.72 ‒ 1.73) | 1.5% | 3.01 (1.72 ‒ 5.27) | 11.0% | 1.62 (1.02 ‒ 1.57) | 16.7% | 1.24 (1.00 ‒ 1.53) |
| SAVR | 5.9% |  | 0.5% |  | 8.8% |  | 13.5% |  |
| All-cause mortality |  |  |  |  |  |  |  |  |
| TAVR | 2.7% | 0.87 (0.65 ‒ 1.16) | 9.1% | 0.94 (0.84 ‒ 1.06) | 16.7% | 0.92 (0.83 ‒ 1.03) | 47.4% | 1.16 (1.08 ‒ 1.26) |
| SAVR | 3.2% |  | 9.8% |  | 18.0% |  | 41.0% |  |
| CV mortality |  |  |  |  |  |  |  |  |
| TAVR | 2.6% | 1.04 (0.71 ‒ 1.51) | 5.6% | 0.91 (0.76 ‒ 1.09) | 9.8% | 0.87 (0.74 ‒ 1.02) | 23.4% | 1.12 (1.00 ‒ 1.26) |
| SAVR | 2.6% |  | 6.2% |  | 11.2% |  | 25.5% |  |
| Stroke |  |  |  |  |  |  |  |  |
| TAVR | 4.1% | 0.82 (0.64 ‒ 1.04) | 5.1% | 0.89 (0.75 ‒ 1.06) | 7.7% | 0.85 (0.71 ‒ 1.02) | 11.43% | 1.18 (0.96 ‒ 1.46) |
| SAVR | 5.0% |  | 5.8% |  | 9.1% |  | 9.7% |  |

Note: NOAF, new-onset atrial fibrillation; TAVR, transcatheter aortic valve replacement; SAVR, surgical aortic valve replacement; MI, myocardial infarction; RF, renal failure. DM, diabetes mellitus; Cr, creatinine; TIA, transient ischemic attacks; PVD, peripheral vascular disease; PM, pacemaker; CAD, coronary artery disease; RR, risk ratio; CI, confidence intervals.
